# Evaluation of penetrating cardiac stab wounds

**DOI:** 10.1186/s13049-015-0190-3

**Published:** 2016-01-22

**Authors:** Mehdi Bamous, Abdou Abdessamad, Jawad Tadili, Ali Kettani, Mamoun Faroudy

**Affiliations:** Department of Cardiac Surgery, Mohamed V Military Hospital, Mohamed V University, Rabat, Morocco; Department of Anesthesiology, Trauma Unit, Ibn Sina Hospital, Mohamed V University, Rabat, Morocco

**Keywords:** Trauma, Cardiac trauma, Penetrating injuries

## Abstract

**Background:**

The purpose of this study was to identify factors associated with unfavourable outcome following stab wounds to the heart in order to improve selection of patients who may benefit from resuscitative effort.

**Methods:**

From February to March, variables were collected from medical records of patients sustaining cardiac trauma. The inclusion criterion was the presence of a penetrating cardiac injury confirmed intraoperatively.

**Results:**

Ninety-eight patients were admitted with penetrating cardiac injury. The mortality rate was 60 %. Fifty-seven patients had unrecordable blood pressure at admission and emergency department thoracotomy was done in twelve patients. The AAST-OIS score was higher in non survivors group (4,21 vs 4,49). Multivariate analysis identified tamponade, associated injuries, right ventricular laceration as the most predictive variables for mortality.

**Discussion:**

Stab wounds should be separated from gunshots wounds as the former mechanism has different pathophysiological issue. Patients arriving without signs of life may benefit from aggressive resuscitative efforts depending on transport time.

**Conclusion:**

Penetrating cardiac injuries are highly lethal condition. Cardiac tamponade, right ventricle lacerations and associated extra-cardiac injuries are independent risk factors of death.

## Background

Stab wounds of the heart represent a significant surgical challenge because of their unpredictable clinical course and the need for emergency clinical care often including emergency room thoracotomy. In the past, the diagnostic workup of patients with suspected cardiac injury was predominantly clinical. However, in the current era, a number of new diagnostic and therapeutic modalities have been introduced in clinically stable patients. Reported outcomes in the literature are widely variable, reflecting a variety of presentation, injury mechanism, prehospital care capabilities and geographic situation. In addition, series are heterogeneous comprising stab and gunshot wounds, these concerns prompted us to review our institutional experience in an attempt to describe and compare the variables associated with patients sustaining penetrating cardiac injuries and identifying risk factors for death.

## Methods

This was a retrospective study undertaken at our trauma unit over a period of 7 years, between February 2005 and March 2012. All patients with penetrating cardiac injuries resulting from stab wounds were included in this study. Penetrating cardiac injury was defined as any injury affecting the pericardium and its content and this injury was confirmed intraoperatively for all patients. We identified patients by diagnosis from the hospital’s trauma registry. The medical records of patients who underwent operation for penetrating cardiac injury were reviewed. The following epidemiologic and trauma related data were carried out: name, age, sex, mechanism of injury, clinical findings on admission, diagnosis, surgical treatment and complications. All study patients had manifested signs of life in the field and/or during transport.

According to the clinical status on admission, we distinguished patients with unrecordable vital signs (unconscious with no detectable pulse and blood pressure), patients with signs of low cardiac output in whom systolic blood pressure (SBP) remains < 90 mmhg and hemodynamically stable patients with SBP > 90 mmhg. The management strategy for all patients presenting in the emergency room with precordial wounds, especially with hypotension and distended neck veins, included immediate central venous access (usually through subclavian vein) rapid crystalloid infusion and insertion of a chest tube on the side of the injury. If the patients remained lifeless and the transferring paramedic didn’t report any recordable signs of life during transport, the patients were intubated and transferred to the operating room with ongoing cardiopulmonary resuscitation (CPR).

For patients who lost their vital signs in the resuscitation room, they underwent emergency department thoracotomy (EDT). Patients with systolic blood pressure less than 90 mmhg were directly transferred to the operating theatre for surgical exploration. Stabilized patients with suspected cardiac injury underwent chest x-ray and transthoracic echocardiography (TTE), any visible fluid or clot in the pericardial space was considered to be positive. Subxyphoid window (SPW) with pericardial decompression has been reserved for stable patients with delayed presentation of traumatic pericardial effusion and for those who undergone a laparotomy with suspiscion of cardiac trauma.

Patients were usually explored via median sternotomy, left anterior thoracotomy was performed for EDT. Injuries of the cardiac chamber were repaired with Teflon mattress suture. Distal coronaries arteries lesions were repaired by simple ligation. Cardiopulmonary bypass wasn’t used in this acute setting. Ethical approval was not required because the study was done retrospectively and all patients were managed according to available guidelines at that moment. A new protocol or medication was not evaluated.

### Statistical analysis

All continuous variables are expressed as mean with standard deviation. The categorical data were expressed as frequency and percentage. Chi square test was applied for comparing categorical variables. The Student Test was used for the comparison of continuous variables. A multiple logistic regression analysis was performed to estimate independent predictive factors for death. This model included risk factors that were first identified by univariate analysis. A p < 0.05 was statistically significant. Data processing and analysis were performed using SPSS for windows, version 13 (SPSS, Inc, Chicago, IL, USA).

## Results

During the 7 years study period, 98 patients with penetrating cardiac injuries were admitted and managed at our trauma unit. Eighty six were males (88 %) and the mean age was 32 ± 14 years . All patients sustained stab wounds. Fifty seven (58 %) had unrecordable blood pressure on arrival with Glascow Coma Scale (GCS) at 3. Twelve (12 %) patients underwent EDT with a mortality rate of 100 %. Fifty three patients (54 %) presented with at least one clinical sign of tamponade, otherwise the diagnosis of tamponade was done intraoperatively. Regarding diagnostic investigations, TTE was performed in17 patients (17 %) with six false negatives, two of them deteriorated and were taken to the operating room and the last four had subxyphoid window that showed large hemopericardium. Median sternotomy was used in 86 patients and left anterior thoracotomy was performed for EDT and in some stable patients. Combined approach was used in four patients. The most frequent injury site was the right ventricle (RV) (52 %), followed by the left ventricle (LV) (38 %), right atrium (RA) (7 %) and left atrium (LA)(3 %). Forty-six patients (47 %) had associated injuries : lung: 18, left internal mammary artery: 8, right internal mammary artery: 6, left anterior descending artery: 5, right coronary artery :2 and intra-abdominal lesions necessitating laparotomy 11: liver : 4, gastric:4, spleen:3 . Laparotomy was done first in 2 patients followed by median sternotomy after the hemopericardium was confirmed by SPW. The AAST-OIS score of the non survivors was significantly higher than for survivors (*p* = o,o3). The mean hospital stay was 10 ± 5 days (Table 1).

**Table 1:** demographic, clinical characteristics

Items	Survivors	Dead	
	*N* = 39	*N* = 59	*P* value
Age (years)*	28,02 ± 12,77	34,02 ± 14,33	0,06
Sexe (M/F)	37/2	49/10	0,08
SBP (mmHg)			
• unrecordable • 0 < SBP <90 • SBP > 90	0	57/ 96,6 %	0001
	22/56,41 %	2/3,38 %	
	17/43,58 %	0	
TPD	34/87,20 %	19/32,20 %	<0001
ERT	0	12/30,8 %	<0,001
AL AAST-OIS	11/28,2 4,21 ± 0,4	33/55,90 % 4,49 ± 0,5	0,007 0,003
Cardiac injury			
• RV	27/69,20 %	24/40,70 %	
• LV	8/20,50 %	29/49,20 %	0,03
• RA	3/7,70 %	4/6,80 %	
• LA	1/ 2,6 %	2 / 3,4 %	

*SBP* systolic blood pressure, *TPD* tamponade, *ERT* emergency room thoracothomy, *AL* associated lesions, *RV* right ventricle, *LV* left ventricle, *RA* right atrium, *LA* left atrium

*mean ± standard deviation

Major complications are hemiparesia in 2 patients and one residual ventricular septal defect in one patient, the defect was hemodynamically significant with impossibility to wean the patient from the ventilator. It was successfully closed on cardiopulmonary bypass at day 2. The mortality rate was (60 %). Concerning clinical variables at hospital admission, SBP < 90mmhg, CPR and GCS score weren’t significant risk factors for mortality in a univariate analysis. However, the presence of tamponade, extra cardiac associated injuries and penetrating injury to the RV were significant risk factors for mortality. Using a multivariate regression analysis, the presence of tamponade was the variable with the highest risk of mortality (OR: 20; *p* < 0001), followed by RV laceration (OR: 10; *p* < 0001) and associated injuries (OR:4; *p* < 0.02) (Table 2).

**Table 2:** Predictive power of studied factor on mortality

	Univariate analysis			Multivariate analysis		
	OR	IC	p	OR	IC	*p*
SBP (mmHg):						
•SBP ≥ 90	1	_	Ref			
•0 < SBP <90	0	[0–3,60 + 139]	0,955			
•unrecordable	0	[0–4,14 + 141]	0891			
TPD	14,31	[4832–42,411]	<0001	20,42	[5336–78,141]	<0001
CPR	3,887,595	[0–8,6 + 56]	0798			
AL	3,23	[1358–7684]	0008	4,27	[1196–15,241]	0025
Cardiac injury :						
•LV	1	_	Ref	1	_	Ref
•RV	4,08	[1567–10,614]	0004	10,620	[2748–41,036]	0001
•RA	2,72	[0502–14,723]	0246	23,214	[1698–317,455]	0018
•LA	1,81	[0145–22,636]	0644	17,151	[0532–552,441]	0109

*SBP* systolic blood pressure, *TPD* tamponade, *AL* associated lesions, *RV* right ventricle, *LV* left ventricle, *RA* right atrium, *LA* left atrium, *Ref* reference

## Discussion

Faced with the rising incidence of violence in our city, penetrating cardiac injuries are commonly encountered and remain highly lethal condition. A review by Campbell et al. found that only 6 % of 1198 patients with penetrating cardiac injury reached the hospital alive [1]. In contrast with this, some studies reported high survival rate up to 90 % but omit the physiologic status upon presentation of these critical patients and they are biased by reporting only patients in good physiologic condition at admission. Thus, this wide variation of mortality, between 10–90 %, reflecting patients selection have contributed to the false perception that the lethality of cardiac injuries has diminished [2–4]. The mortality rate in our study group was 60 %, explained by the fact that the majority of patients have pre-hospital factors predictive of poor outcome such as absence of vital signs during transfer, dilated pupils and absence of motion in the extremities. The protective effect attributed by some authors to tamponade hasn’t been observed in this series [5]. However, we found the presence of pericardial tamponade to be a critical independent factor predictive of mortality in a multivariate analysis. Our finding is consistent with the result of three prospective studies which didn’t find any protective effect of cardiac tamponade after penetrating cardiac injuries [6–8]. It seems that a slow and gradual haemorrhage is much better tolerated as it can be gradually accommodated by the pericardium but any acute rises in intrapericardial pressure or any delay in intervention resulted in a adverse effect in cardiac function. We reserved EDT for patients arresting at admission in the emergency room . For this indication, we observed 100 % mortality. Similarly, many reports showed the same mortality rate and suggested that EDT must be abandoned because it’s ineffective, expensive and high risky procedure [9, 10]. Due to the lack of evidence-based guideline for the indication of the EDT, the western trauma association committee analysed the available studies in the literature to define a rational approach for the appropriate role of the EDT. They found that the succes of performing EDT approximated 35 % for patient arriving in shock caused by penetrating cardiac injury. Conversely, this survival rate is less than 1 % for patients without vital signs on admission. They recommended that EDT was best indicated for patients with penetrating injury when prehospital CPR was less than 15 minutes and in patients with refractory shock [11].

As prehospital care system develop and as patients are subjected to a greater degrees of violence, we are facing to manage patients with multiple associated injuries. In this setting, thoracotomy and laparotomy are required at the same time to deal with injuries in both cavities. Thus, clinical decision making is crucial and priority should be given to the lesions causing the greatest blood loss. Subxyphoid windows is considered a reliable diagnostic tool by confirming the presence of a hemopericardium, in this subset of patients [12]. We used SPW in 2 patients who have to undergo laparotomy with 100 % sensitivity and we found associated injuries an independent predictive factor of mortality. Various scores exist to estimate the severity of cardiac injury. The lack of uniformity of protocols utilised coupled with the use of physiologic and anatomic parameters which have not been statistically validated regarding their predictive values render these scores quite difficult to interpret. Mortality for AAST-OIS grade 5 is higher than grade 4, so a correlation between survivors and non survivors regarding mortality and higher AATS-OIS score was validated in this study.

Our study, despite investigating outcomes in a large and homogenous material resulting from a single type of cardiac injury, still suffer from missing data like a tranport time and duration of CPR before admission. While we identified, by multivariate analysis, reliable predictors of mortality these missing variables prevented us to find any single preoperative factor that predict mortality. As has been pointed out previously by the authors [11], no controlled randomized trial exists to select patients who may or may not benefit from aggressive rescussitative effort according to prehospital variables and current recommendations concerning aggressive rescussitative effort are based only on retrospective studies and on expert opinion. In our institution we still prefer exploring our patients arriving without signs of life by sternotomy because it offers a good access to the heart, great vessels and both pleural spaces. All heart surfaces are easily explored and we can extend it to the abdomen to control intraperitoneal bleeding. When it’s performed by a cardiac surgeon it’s done rapidly without wasting time. For EDT, it’s reserved for patients arresting in the emergency room with the goal to stop haemorrhage while transferring the patient to the operating theatre for further exploration.

## Conclusion

Unlike other studies, this review examines stab wounds separately from gunshots wounds as the latter mechanism has different pathophysiology and outcome. We found that cardiac tamponade, right ventricle lacerations and associated extra-cardiac injuries are independent risk factors of death. A systematic approach to these patients is necessary so that other life-threatening lesions are treated appropriately and in a timely fashion.
